# Potency preservation following stereotactic body radiation therapy for prostate cancer

**DOI:** 10.1186/1748-717X-8-256

**Published:** 2013-11-01

**Authors:** Olusola Obayomi-Davies, Leonard N Chen, Aditi Bhagat, Henry C Wright, Sunghae Uhm, Joy S Kim, Thomas M Yung, Siyuan Lei, Gerald P Batipps, John Pahira, Kevin G McGeagh, Brian T Collins, Keith Kowalczyk, Gaurav Bandi, Deepak Kumar, Simeng Suy, Anatoly Dritschilo, John H Lynch, Sean P Collins

**Affiliations:** 1 Department of Radiation Medicine, Georgetown University Hospital, 3800 Reservoir Road, NW, Washington, DC 20007, USA; 2 Department of Urology, Georgetown University Hospital, Washington, DC, USA; 3 Department of Biology, Cancer Research Laboratory, University of the District of Columbia, Columbia, Washington, DC, USA

**Keywords:** Prostate cancer, SBRT, CyberKnife, EPIC, Bother, Potency, Penile bulb

## Abstract

**Background:**

Erectile dysfunction after prostate radiation therapy remains an ongoing challenge and critical quality of life issue. Given the higher dose of radiation per fraction using stereotactic body radiation therapy (SBRT) there is concern that post-SBRT impotency would be higher than conventional radiation therapy approaches. This study sought to evaluate potency preservation and sexual function following SBRT for prostate cancer.

**Methods:**

Between February 2008 and March 2011, 216 men with clinically localized prostate cancer were treated definitively with SBRT monotherapy at Georgetown University Hospital. Potency was defined as the ability to have an erection firm enough for intercourse with or without sexual aids while sexual activity was defined as the ability to have an erection firm enough for masturbation and foreplay. Patients who received androgen deprivation therapy (ADT) were excluded from this study. Ninety-seven hormone-naïve men were identified as being potent at the initiation of therapy and were included in this review. All patients were treated to 35–36.25 Gy in 5 fractions delivered with the CyberKnife Radiosurgical System (Accuray). Prostate specific antigen (PSA) and total testosterone levels were obtained pre-treatment, every 3 months for the first year and every 6 months for the subsequent year. Sexual function was assessed with the Sexual Health Inventory for Men (SHIM), the Expanded Prostate Index Composite (EPIC)-26 and Utilization of Sexual Medication/Device questionnaires at baseline and all follow-up visits.

**Results:**

Ninety-seven men (43 low-, 50 intermediate- and 4 high-risk) at a median age of 68 years (range, 48–82 years) received SBRT. The median pre-treatment PSA was 5.9 ng/ml and the minimum follow-up was 24 months. The median pre-treatment total serum testosterone level was 11.4 nmol/L (range, 4.4-27.9 nmol/L). The median baseline SHIM was 22 and 36% of patients utilized sexual aids prior to treatment. Although potency rates declined following treatment: 100% (baseline); 68% (6 months); 62% (12 months); 57% (18 months) and 54.4% (24 months), 78% of previously potent patients had erections sufficient for sexual activity at 24 months post-treatment. Overall sexual aid utilization increased from 36% at baseline to 49% at 24 months. Average EPIC sexual scores showed a slow decline over the first two years following treatment: 77.6 (baseline); 68.7 (6 months); 63.2 (12 months); 61.9 (18 months); 59.3 (24 months). All sexual functions including orgasm declined with time. Prior to treatment, 13.4% of men felt their sexual function was a moderate to big problem which increased to 26.7% two years post treatment. Post-treatment testosterone levels gradually decreased with a median value at two year follow-up of 10.7 nmol/L. However, the average EPIC hormonal scores did not illustrate a statistically significant difference two years post-treatment. Review of the radiation doses to the penile bulb in this study, a potential marker of post-treatment sexual function, revealed that the dose was relatively low and at these low doses the percentage of the penile bulb receiving 29.5 Gy did not correlate with the development of ED.

**Conclusions:**

Men undergoing SBRT monotherapy for prostate cancer report sexual outcomes comparable to those reported for conventional radiation modalities within the first 24 months after treatment. Longer follow-up is required to confirm the durability of these findings.

## Background

Post-treatment sexual function is a primary determinant of satisfaction following prostate radiotherapy [1,2]. Erectile dysfunction (ED) occurs commonly following external beam radiation therapy (EBRT) and/or brachytherapy [3]. The incidence of ED is dependent on the potency definition [4,5] and on the manner of data collection (i.e. patient or physician reported) [6]. Patients with radiation-induced impotence report a decrease in the quality and reliability of erection, ability to reach orgasm and overall ability to function sexually [7]. ED develops months to years after radiation therapy without recovery [2]. Patient characteristics such as a history of baseline ED [8], older age [9], obesity, comorbidities and pretreatment sexual aid usage may increase an individual’s risk of radiation induced ED [10]. In addition, utilization of androgen deprivation therapy (ADT) [2] and radiation-induced hypogonadism [11] can adversely affect sexual outcomes while post-treatment utilization of sexual aids may enhance them [12-14].

The etiology of radiation induced ED is currently unclear but may involve damage to the neurovascular bundles (NVBs) [15], crura [16,17] and/or penile bulb (PB) [16,18]. Thus treatment-related factors such as the radiation dose to these adjacent sexual structures may contribute to the incidence and severity of ED. The risk of ED may be related to the volume of the crura/penile bulb in the high dose area [16]. To minimize ED, it is currently recommended that the median dose to the penile bulb be limited to < 50 Gy with conventionally fractionated EBRT [19]. Studies have shown that advanced radiation technologies such as intensity modulated radiation therapy (IMRT) may decrease the dose to the crura/penile bulb and potentially decrease ED [20,21].

The optimal radiation schedule for the treatment of prostate cancer is under active clinical investigation. Recent data suggest that large radiation fraction sizes are radiobiologically favorable over lower fraction sizes in prostate cancer [22,23]. The α/β for prostate cancer may be as low as 1.5 Gy [22]. If the α/β for prostate cancer is less than 3 Gy, which is generally the value accepted for late sexual complications, the linear-quadratic model predicts that delivering large radiation fraction sizes will result in improved local control with a similar rate of sexual function complications.

SBRT is a safe and effective treatment for clinically localized prostate cancer [24-26]. The use of large fraction sizes in SBRT offers the potential radiobiological benefits of hypofractionation and potentially may minimize radiation-associated ED by reducing the volume of critical structures receiving high radiation doses. The CyberKnife robotic radiosurgical system uses image guidance to track implanted fiducials to account for intrafraction prostatic motion [27,28]. This reduces the uncertainty of the location of the prostate and allows treatment to be delivered with a smaller treatment volume expansion, which may reduce the doses delivered to the adjacent sexual structures. Initial reports suggest that the incidence of ED following SBRT is comparable to other radiotherapy modalities [29]. The goal of this study is to report the sexual outcomes following SBRT for clinically localized prostate cancer in previously potent men.

## Methods

### Patient selection

Patients eligible for this study were those who had histologically-confirmed prostate cancer and were potent (patient-reported response of “firm enough for intercourse” on Question 9 of the baseline EPIC-26) with or without sexual aids prior to treatment. Patients who received ADT were excluded from this study. Institutional IRB approval was obtained for retrospective review of data that was prospectively collected in our institutional database.

### SBRT treatment planning and delivery

SBRT treatment planning and delivery were conducted as previously described [30]. Briefly, gold fiducials were placed into the prostate. Fused CT and MR images were used for treatment planning. The clinical target volume (CTV) included the prostate and the proximal seminal vesicles. The planning target volume (PTV) equaled the CTV expanded 3 mm posteriorly and 5 mm in all other dimensions. The prescription dose was 35–36.25 Gy to the PTV delivered in five fractions of 7–7.25 Gy over one to two weeks. The PB (proximal portion of the corpus spongiosum) was contoured [31] and evaluated with dose-volume histogram analysis during treatment planning using Multiplan (Accuray Inc., Sunnyvale, CA) inverse treatment planning. The dose-volume histogram (DVH) goal was for < 50% PB volume receiving 29.5 Gy. Assuming an α/β of 3 Gy for late sexual complications, this is biologically equivalent to approximately 50 Gy administered in 2 Gy fractions [32]. To minimize the risk of local recurrence, the dose to the NVBs was not restrained. Target position was verified during treatment using paired, orthogonal x-ray images.

### Follow-up and statistical analysis

Prostate-specific antigen (PSA) and total testosterone levels were obtained before treatment and during routine follow-up visits every 3 months for the first year and every six months for the second year. Sexual function was assessed with the Expanded Prostate Index Composite (EPIC)-26 [7] and Utilization of Sexual Medication/Device questionnaires at baseline and at all follow-ups. The EPIC-26 sexual function domain includes five questions related to sexual function and one question related to sexual bother (degree of interference or annoyance caused by limitations in sexual function) [33]. The sexual functions assessed included quality of erection (potency), ability to have erection, reliability of erections, ability to reach orgasm and overall ability to function sexually. The definition of potency and sexual activity were based on the patient-reported response to Question 9 on the EPIC-26. Potency was defined as the ability to have an erection firm enough for intercourse [34] while sexual activity was defined as the ability to have an erection firm enough for masturbation and foreplay [4].

Student’s t-test and chi-square analysis were used to assess differences in ongoing PSA, testosterone and quality of life scores in comparison to baseline. Sample medians and ranges were used to describe continuous variables including PSA and testosterone. For each EPIC question, the responses were grouped into two to three clinically relevant categories. To statistically compare changes between time points, the levels of responses were assigned a score and the significance of the mean changes in the scores was assessed by paired *t* test. EPIC scores for the sexual domain and its individual questions range from 0–100 with lower values representing worsening sexual symptoms. The minimally important difference (MID) in EPIC score was defined as a change of one-half standard deviation (SD) from the baseline [35]. To limit the effect of attrition bias, statistical analysis was limited to time points in which ≥ 80% of the patient data were available.

The impact of baseline patient characteristics on potency rates two years post-SBRT were evaluated by univariate and multivariate analyses. Univariate analysis of variance (ANOVA) was used to detect significant relationship between patient characteristics and potency at 2 years post treatment. In multivariate analysis, stepwise ordinal logistic regression modeling was used to determine independent factors predicting sexual function outcome. The baseline patient characteristics that were included as variables in the univariate and multivariant analyses included age, race, partner status, comorbidity, body mass index (BMI), risk group, work status, Sexual Health Inventory for Men (SHIM) score, erectile function and sexual aid usage. All tests were two-tailed, and a *p* value <0.05 was considered significant. IBM® SPSS version 21 and MedCalc® version 12.6.1.0 were used to perform the statistical analyses.

## Results

From February 2008 to March 2011, 216 prostate cancer patients were treated per our institutional SBRT monotherapy protocol. Ninety-seven men who were identified as being potent at the initiation of therapy and had a minimal follow up of two years post-treatment were included in this review (Table 1). They were ethnically diverse with a median age of 66.8 years (range, 48–82 years). The median pre-treatment total serum testosterone level was 11.4 nmol/L (range, 4.4 - 27.9 nmol/L). 43 patients were low-, 50 intermediate-, and 4 high-risk. 49.5% patients had ED prior to treatment (baseline SHIM ≤ 21) with a median baseline SHIM of 22 (range, 3–25). 36.1% of patients were utilizing sexual aids prior to SBRT. Phosphodiesterase 5 (PDE5) inhibitors were utilized most commonly (36%) followed by vacuum devices (1%). No patients utilized transurethral suppositories or penile injections prior to treatment.

**Table 1:** Baseline patient characteristics and treatment

**Patients (n = 97)**		
**Age (years)**	Age ≤ 60	14 (14.4%)
	60 < Age ≤ 70	55 (56.7%)
	Age > 70	28 (28.9%)
**Race**		
	White	47 (48.5%)
	Black	46 (47.4%)
	Other	4 (4.1%)
**Median Pre-Tx PSA (ng/mL) Median**		5.9 (1.8-32.5)ng/mL
**Median Pre-Tx testosterone (nmol/L)**		11.4 (4.4-27.9)nmol/L
**Partner status**		
	Partnered	73 (75.3%)
	Not partnered	24 (24.7%)
**Charlson comorbidity index (CCI)**		
	0	78 (80.4%)
	1	13 (13.4%)
	2	5 (5.2%)
	3	1 (1%)
**Body mass index (BMI)**		
	<25	24 (24.7%)
	25-29.99	46 (47.4%)
	30-34.99	23 (23.7%)
	≥ 35	4 (4.1%)
**Risk group (D’ Amico’s)**		
	Low risk	43 (44.3%)
	Intermediate risk	50 (51.5%)
	High risk	4 (4.1%)
**Work status**		
	Working	57 (58.8%)
	Non-working	40 ((41.2%)
**Pre**-**treatment SHIM score**		
	22-25 (No ED)	49 (50.5%)
	17-21 (Mild ED)	32 (33.0%)
	12-16 (Mild Moderate ED)	10 (10.3%)
	8-11 (Moderate ED)	2 (2.1%)
	< 8 (Severe ED)	^*(No Sexual Activity)^ 4 (4.1%)
**Sexual aid**		
	None	62 (63.9%)
	Oral	35 (36.1%)
	Vacuum	1 (1.0%)
	Suppository/injection	0 (0%)
**Dose (Gy)**		
	36.25	87 (89.7%)
	35	9 (9.3%)
	Other	1 (1.0%)

Ninety percent of patients were treated with 36.25 Gy in five 7.25 Gy fractions (Table 1). The median follow-up was 2.7 years. The median pre-treatment PSA of 5.9 ng/ml declined to a median two years post-treatment of 0.5 ng/ml. There was one biochemical failure, occurring in an intermediate-risk patient. The overall two-year actuarial biochemical relapse free survival was 99%. No patient initiated ADT in the first two years following therapy.

Erections sufficient for intercourse declined following treatment: 100% (baseline); 67.8% (6 months); 62.2% (12 months); 57% (18 months) and 54.4% (24 months) (Table 2) which were statistically significant (*p* < 0.0001) at all time points. The decline in potency at two years was unlikely due solely to aging, as the average age of potent patients (66.1 y/o) was not statistically different from non-potent patients (68.0 y/o) (*p* = 0.17). However, at two year post-SBRT, 77.8% of patients had erections that were satisfactory for sexual activity including masturbation and foreplay (Table 2). A Charlson Comorbidity Index of ≥ 1 was significantly associated with a decreased probability of potency preservation at two years on univariate (*p* = 0.007) , but was not significant on multivariate analysis (*p* = 0.246) (Table 3). Sexual aid usage prior to SBRT was associated with an increased probability of potency preservation two years following treatment on both univariant (*p* = 0.010) and multivariant analysis (*p* = 0.037) (Table 3). No other baseline patient characteristics were significantly associated potency at two years following SBRT. Two years post-treatment, overall sexual aid utilization increased to 48.9%: oral medications (48.9%), vacuum device (6.7%), urethral suppository (2.2%) and penile injection (2.2%) (Table 4). No men utilized a penile prosthesis.

**Table 2:** **Quality of erection following SBRT for prostate cancer** (**patient**-**reported responses to question 9 of the EPIC**-**26**)

**Follow up (months)**	**Start**	**3**	**6**	**9**	**12**	**18**	**24**
Firm enough for intercourse	100.0%	73.7%	67.8%	66.7%	62.2%	57.0%	54.4%
Firm enough for masturbation and foreplay only	0.0%	9.5%	18.4%	17.2%	17.8%	26.6%	23.3%
Not firm for any sexual activity	0.0%	16.8%	13.8%	16.1%	20.0%	16.5%	22.2%
*p*		<0.0001	<0.0001	<0.0001	<0.0001	<0.0001	<0.0001
Total patient	97	95	87	87	90	79	90

**Table 3:** **Impact of baseline patient characteristics on potency rates two years post**-**SBRT**

**Characteristics**		**% Potent 2 years after SBRT**	***p *** **value**
**Age**			
	≤ 65 y/o pre-RT	66.7%	0.580
	>65 y/o pre-RT	47.4%	
**Race**			
	White	51.2%	0.574
	Non-white	57.4%	
**Partner status**			
	Partnered	54.3%	0.297
	Non-partnered	55.0%	
**Charlson comorbidity index (CCI)**			
	0	58.9%	0.007*
	≥1	35.3%	
**Body mass index (BMI)**			
	< 30	60.9%	0.943
	≥ 30	38.5%	
**Risk group (D’ Amico’s)**			
	Low risk	54.8%	0.420
	Intermediate-High Risk	54.2%	
**Work status**			
	Working	54.7%	0.173
	Non-working	54.1%	
**Pre-treatment SHIM**			
	≥ 22	40.91%	0.127
	< 22	67.39%	
**Sexual aid**			
	None	43.8%	0.010*^#^
	Yes	60.3%	
**SBRT Dose**			
	36.25 Gy	50.0%	0.933
	< 36.25 Gy	55.0%	

*Significant in univariate analysis; # Significant multivariate analysis.

**Table 4:** Sexual aid utilization following SBRT for prostate cancer

**Sexual aid:**	**Oral**	**Vacuum**	**Suppository**	**Injection**
**Follow-up**				
**Start**	36.1%	1.0%	0.0%	0.0%
**3 mon**	35.8%	1.1%	1.1%	0.0%
**6 mon**	37.9%	2.3%	0.0%	0.0%
**9 mon**	39.1%	2.3%	0.0%	1.1%
**12 mon**	40.0%	4.4%	1.1%	0.0%
**18 mon**	45.0%	3.80025	1.3%	1.3%
**24 mon**	48.9%	3.8%	2.2%	2.2%

**Figure 1:**
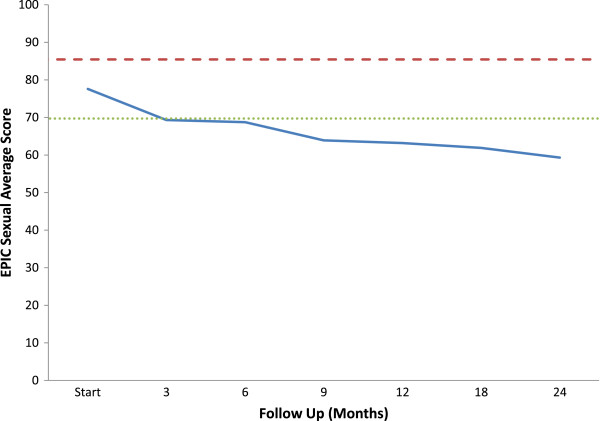
**Average EPIC sexual domain scores at baseline and following SBRT for prostate cancer.** Thresholds for clinically significant changes in scores (½ standard deviation above and below the baseline) are marked with dashed lines. EPIC scores range from 0–100 with higher values representing a more favorable health-related QOL.

**Figure 2:**
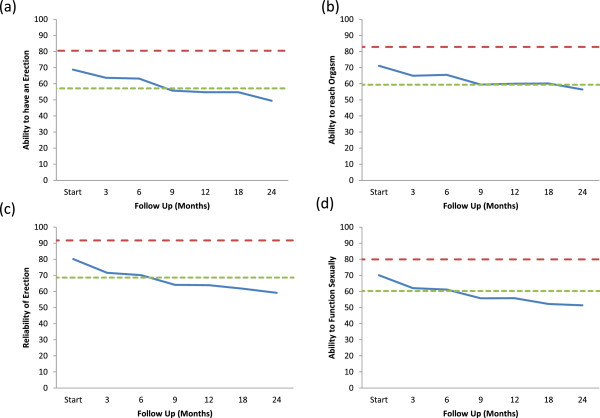
**Average individual EPIC sexual function scores at baseline and following SBRT for prostate cancer. (a)** ability to have an erection-Question 8A of the EPIC-26; **(b)** ability to reach orgasm- Question 8B of the EPIC-26; **(c)** reliability of erection- Question 10 of the EPIC-26; **(d)** ability to function sexually- Question 11 of the EPIC-26. Thresholds for clinically significant changes in scores (½ standard deviation above and below the baseline) are marked with dashed lines. EPIC scores range from 0–100 with higher values representing a more favorable health-related QOL.

Adequate sexual function involves more than just possessing adequate erections [36]. The EPIC sexual domain allows for a more comprehensive and reliable assessment of a patients overall sexual function [37]. Average EPIC sexual scores showed a slow decline over the first two years following treatment: 77.6 (baseline); 68.7 (6 months); 63.2 (12 months); 61.9 (18 months); 59.3 (24 months) (Figure 1). At two year post-treatment, this change was statistically (p < 0.001) and clinically significant (Figure 1). All sexual functions including orgasm declined with time in a similar manner (Figure 2a, d, Table 5).

**Table 5:** **Sexual functions following SBRT for prostate cancer**: **patient**-**reported responses to EPIC**-**26 questions 8A** (**ability to have an erection**), **8B** (**ability to reach orgasm**), **10** (**reliability of erections**) **and 11** (**ability to function sexually**)

	**Start**	**3**	**6**	**9**	**12**	**18**	**24**
**Ability to have an erection**							
Very good-good	58.8%	51.6%	56.3%	43.7%	45.6%	41.8%	36.7%
Fair	34.0%	33.7%	26.4%	26.4%	25.6%	29.1%	31.1%
Poor, very poor and none	7.2%	14.7%	17.2%	29.9%	28.9%	29.1%	32.2%
*p*		<0.04	<0.140	<0.001	<0.001	<0.001	<0.001
**Ability to reach orgasm**							
Very good-good	74.2%	56.8%	63.2%	51.7%	53.3%	53.2%	46.7%
Fair	18.6%	27.4%	18.4%	24.1%	24.4%	22.8%	25.6%
Poor, very poor and none	7.2%	15.8%	18.4%	24.1%	22.2%	24.1%	27.8%
*p*		<0.05	0.089	<0.003	<0.001	<0.001	<0.001
**Reliability of erections**							
More than half-half the time	78.4%	66.3%	64.4%	55.2%	57.8%	54.4%	52.2%
Less than half the time	21.6%	33.7%	35.6%	44.8%	42.2%	45.6%	47.8%
*p*		<0.004	<0.003	<0.001	<0.001	<0.001	<0.001
**Function sexually (overall)**							
Very good-good	63.9%	53.7%	52.9%	47.1%	47.8%	43.0%	40.0%
Fair	33.0%	29.5%	28.7%	24.1%	25.6%	24.1%	30.0%
Poor-very poor	3.1%	14.7%	16.1%	28.7%	26.7%	32.9%	30.0%
*p*		<0.003	<0.005	<0.001	<0.001	<0.001	<0.001
**N=**	**97**	**95**	**87**	**87**	**90**	**79**	**90**

**Figure 3:**
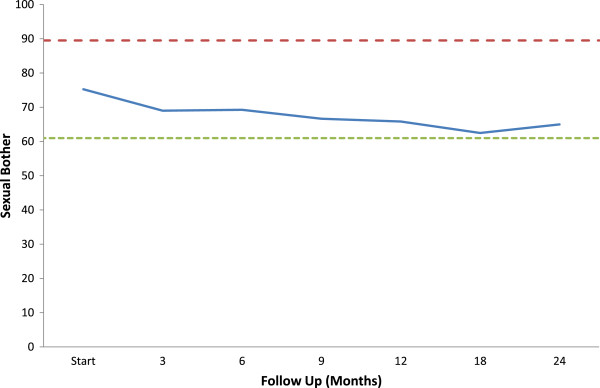
**EPIC sexual bother at baseline and following SBRT for prostate cancer- Question 12 of the EPIC-26.** Average sexual bother scores. Thresholds for clinically significant changes in scores (½ standard deviation above and below the baseline) are marked with dashed lines. EPIC scores range from 0–100 with higher values representing a more favorable health-related QOL.

Sexual bother may be more important to an individual patient than sexual function. As for the overall EPIC sexual domain scores, post-SBRT sexual bother scores were significantly lower than pre-SBRT scores at all time points (Figure 3, Table 6). The proportion of men feeling that their sexual function was a moderate to big problem increased from 13.4% to 30% at 18 months post-SBRT (Figure 3, Table 6).

**Table 6:** Sexual brother following SBRT for prostate cancer (patient-reported responses to question 12 of the EPIC-26)

**Sexual brother**	**Start**	**3 mon**	**6 mon**	**9 mon**	**12 mon**	**18 mon**	**24 mon**
No problem	47.42%	40.86%	42.53%	37.93%	41.11%	41.25%	38.89%
Very small-small problem	39.18%	40.86%	34.48%	40.23%	36.67%	28.75%	34.44%
Moderate-big problem	13.40%	18.28%	22.99%	21.84%	22.22%	30.00%	26.67%

**Figure 4:**
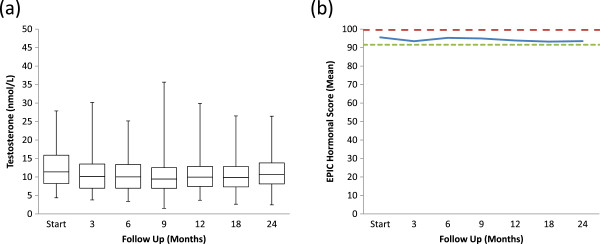
**Pre- and post-treatment testosterone levels and EPIC hormonal scores. (a)** Box**-**and**-**Whisker plot of total testosterone levels. The *p* values were from χ^2^-analysis with baseline testosterone levels. **(b)** Average EPIC hormonal scores. Thresholds for clinically significant changes in scores (½ standard deviation above and below the baseline) are marked with dashed lines. EPIC scores range from 0–100 with higher values representing a more favorable health-related QOL.

Pre-treatment total serum testosterone levels ranged from 4.4 nmol/L to 27.9 nmol/L with a median value of 11.4 nmol/L. 24.7% of patients were hypogonadal (total serum testosterone level below 8 nmol/L) prior to SBRT. At two years the median serum testosterone value of 10.7 nmol/L (range, 2.5- 26.4 nmol/L) was not significantly lower than the pre-treatment value (*p* = 0.07) (Figure 4a). The median absolute reduction was small (0.7 nmol/L) and the median percent reduction was 5.8%. The pre-treatment and 2-year post SBRT biochemical hypogonadism rates were not significantly different (data not shown). At one month post-treatment, the mean EPIC hormone score declined to 93.5 from a baseline of 95.6 (*p* = 0.04); it returned to baseline by 6 months post-treatment (Figure 4b).

Similar to prior reports, the median PB volume in this series was 11 cc [31]. The mean percentage of the PB receiving 29.5 Gy was 7.0% (range: 0–32.7%), with the potent and impotent groups receiving 6.3% (SD 6.5%) and 7.2% (SD 8.6%), respectively. The four patients who received 29.5 Gy to > 25% of the PB were impotent two years following treatment.

## Discussion

Erectile dysfunction following prostate radiation therapy remains an ongoing challenge and critical quality of life issue [1]. Currently, there is limited data on sexual outcomes following SBRT for prostate cancer [29]. A better understanding of the sexual declines following SBRT would enable clinicians to provide more realistic expectations to patients as they weigh the complex treatment options [10]. Our prior report on sexual function following SBRT [30] utilized the SHIM questionnaire which primarily focuses on erectile function and is an inadequate measure of overall sexual function [5,36]. In this study, we utilized the EPIC-26 sexual function domain to evaluate erectile function but also assess overall sexual function including orgasmic function and sexual bother.

For this analysis, potency was rigorously defined as the ability to have an erection firm enough for intercourse [4]. Forty five percent of our patients were potent prior to SBRT and these patients were included in this analysis. Still, they were elderly with relatively low baseline SHIM scores and high pretreatment sexual aid utilization (Table 1). Even so, two years post-SBRT, 54% of these patients remained potent and 78% maintained an erection firm enough for masturbation and foreplay. As seen previously, the greatest decline in potency following radiation therapy occurred in the first year with stabilization afterwards [38]. These results are comparable to results with conventionally fractionated EBRT or brachytherapy [2,10,29].

The etiology of this early decline in potency is unclear but may involve exposure of the NVBs, crura or PB to excessive radiation. In this series, which included patients with unfavorable cancers, there was no attempt to spare the NVBs due to the concern that this may cause an unacceptably high rate of local failures [39]. Post-treatment sexual dysfunction may be exacerbated by high PB doses. With the aim of improving potency, we have modified our institutional protocol. To reduce the PB dose, we have restricted the PB dose to < 25% of the volume receiving 29.5 Gy. Utilizing SBRT, this was easily achievable. PB doses below this did not correlate with impotence. We believe that such modifications have the potential to reduce the incidence and severity of impotence without increasing the risk of local failure.

Declining potency following SBRT suggests a need for approaches to improving long-term sexual outcomes. In this study, pre-treatment sexual aid usage was associated with increased potency two years following SBRT. Sexual aid utilization increased from a baseline of 37% to 49% at 24 months. Oral medication usage was common but vacuum device, urethral suppository, penile injection and/or penile prosthesis were rarely utilized. It is not clear why post-treatment utilization of these potentially effective therapies [10] was not higher. Possible explanations include patient’s perception of potential discomfort, the high cost of such treatments or partner availability. Others have shown that while sexual function may be improved by the use of sexual aids, sexual bother may be enhanced [33].

Like other treatment modalities, this study shows that SBRT impacts all areas of sexual function including erections, orgasm and overall satisfaction [40,41]. For example, only 46.7% of men two years following SBRT reported at least a good ability to reach orgasm compared to 74% prior to treatment (Table 5). The etiology of this decline in the ability to obtain orgasm is unclear but could be related to hemospermia, reduced ejaculate and/or painful ejaculation [42-45]. To better assess the etiology of orgasmic dysfunction, our future studies will employ an instrument to assess the effect of SBRT on ejaculation [46].

Bother may be a better indicator than function on how a treatment impacts an individual patient’s quality of life [47]. Sexual bother is defined as the degree of interference or annoyance caused by limitations in sexual function and is dependent on an individual’s pre-treatment erectile function, libido and partner status [33]. In general, previously potent men report a higher rate of bother following treatment than impotent men [48]. In this series of potent men, sexual bother slowly increased during the first two years following SBRT treatment (Figure 3, Table 6). Similar to other radiation modalities, this rate plateaued at 12–24 months following treatment with 30-40% of men reporting sexual bother as a moderate to big problem [2,49]. As in other series, sexual bother scores were better than sexual function scores.

Our study has several limitations. The penile bulb may not be the critical component of the erectile apparatus, but may be a surrogate for the crura in conventional radiation therapy treatment planning [16]. The utilization of non-coplanar beams in SBRT treatment could yield unexpectedly high doses to the crura [50]. Future studies should determine the crura dose during SBRT treatment and its impact on post-SBRT potency. In addition, the pretreatment utilization of sexual aids in our patients was high compared to previous reports [29]. The etiology is unclear but may limit the generalizability of this study.

## Conclusions

SBRT was well-tolerated for these patients with clinically localized prostate cancer. Men with preserved potency undergoing SBRT monotherapy for prostate cancer have sexual outcomes comparable to those reported for alternative radiation modalities within the first 24 months after treatment. Although most men retained sexual function, declining potency following treatment suggest a need for new approaches to improve long-term sexual outcomes.

## Abbreviations

ADT: Androgen deprivation therapy; CTV: Clinical target volume; DVH: Dose-volume histogram; GTV: Gross target volume; NVBs: Neurovascular bundles; PB: Penile bulb; PTV: Planning target volume; QoL: Quality of life; SHIM: Sexual health inventory for men; EBRT: External beam radiation therapy; SBRT: Stereotactic body radiation therapy; EPIC: Expanded prostate index composite.

## Competing interests

SP Collins and BT Collins serve as clinical consultants to Accuray Inc. The other authors declare that they have no competing interests.

## Authors’ contributions

OO and LC are lead authors, who participated in data collection, data analysis, manuscript drafting, table/figure creation and manuscript revision. AB aided in the quality of life data collection and maintained the patient database. HW aided in the quality of life data collection and statistical analysis. SU aided in the quality of life data collection. JK aided in the quality of life data collection and maintained the patient database, aided in data collection, and participated in initial data interpretation. TY aided in the quality of life data collection and clinical data collection. SL is the dosimetrist who developed the majority of patients’ treatment plans, and contributed to the dosimetric data analysis and interpretation. GB, JP, and KM aided in clinical data collection. BC, KK, GB, and DK participated in the design and coordination of the study. SS aided in statistical analysis, quality of life analysis and manuscript revision. AD is a senior author who aided in drafting the manuscript. JL is a senior author who aided in drafting the manuscript. SC was the principal investigator who initially developed the concept of the study and the design, aided in data collection, drafted and revised the manuscript. All authors read and approved the final manuscript.
